# Effects of *Litchi chinensis *fruit isolates on prostaglandin E_2 _and nitric oxide production in J774 murine macrophage cells

**DOI:** 10.1186/1472-6882-12-12

**Published:** 2012-03-01

**Authors:** Yang Zhou, Hong Wang, Ruili Yang, Hui Huang, Yuanming Sun, Yudong Shen, Hongtao Lei, Hong Gao

**Affiliations:** 1 Guangdong Provincial Key laboratory of Food Quality and Safety, College of Food Science, South China Agricultural University, Wushan Road, Guangzhou 510642, China

## Abstract

**Background:**

*Litchi chinensis *is regarded as one of the 'heating' fruits in China, which causes serious inflammation symptoms to people.

**Methods:**

In the current study, the effects of isolates of litchi on prostaglandin E_2 _(PGE_2_) and nitric oxide (NO) production in J774 murine macrophage cells were investigated.

**Results:**

The AcOEt extract (EAE) of litchi was found effective on stimulating PGE_2 _production, and three compounds, benzyl alcohol, hydrobenzoin and 5-hydroxymethyl-2-furfurolaldehyde (5-HMF), were isolated and identified from the EAE. Benzyl alcohol caused markedly increase in PGE_2 _and NO production, compared with lipopolysaccharide (LPS) as positive control, and in a dose-dependent manner. Hydrobenzoin and 5-HMF were found in litchi for the first time, and both of them stimulated PGE_2 _and NO production moderately in a dose-dependent manner. Besides, regulation of cyclooxygenase-2 (COX-2) and inducible nitric oxide synthase (iNOS) mRNA expression and NF-κB (p50) activation might be involved in mechanism of the stimulative process.

**Conclusion:**

The study showed, some short molecular compounds in litchi play inflammatory effects on human.

## Background

Prostaglandin E_2 _is the major prostaglandin produced by macrophage cells, which is derived from 20-carbon polyunsaturated fatty acids synthesized in a wide range of tissue types. In humans, PGE_2 _is a well-known pro-inflammatory mediator involved in diverse functions, including heat releasing, strengthening the permeability of blood vessel, accelerating the release of lysozyme, interferon, erythrogenin, colony stimulating factor and accelerating chemotaxis of leucocyte et al. [1]. The key regulatory enzyme of PGE_2 _biosynthesis is cyclooxygenase, and cyclooxygenase-2 is the inducible form of this enzyme and mediates many of the inflammatory and inducible effects. It is therefore worth screening the effect of foods on COX-2 expression and PGE_2 _formation which can provide useful information on inflammatory conditions [2].

Nitric oxide is known to play an important role in the maintenance of tissue homeostasis. NO is produced by nitric oxide synthase, and the inducible isoform (iNOS) is known to be implicated under several pathological conditions including inflammation. NO produced by iNOS kills infectious pathogens, but Overproduction of NO result damage to tissues and eventually destruction of tissue homeostasis [3]. Thus, the iNOS expression and NO production might be a good target for inflammatory research.

For 2,000 years, people in Chinese society have believed that certain foods are either 'heating' (or 'fire increasing') or 'cooling' (or 'fire reducing') in the body when eaten [4]. According to traditional Chinese medicine, symptoms for the diagnosis of a 'heating' disease state include dry mouth, a bitter taste sensation, throat swelling and sore, boil of the lips and a rough yellow tongue, flushing and even fever [5].

In contrast, disease states in which the patient is thirsty but is not willing to drink, and has pallor, diarrhea, a large amount of diluted urine, a smooth white tongue and a slow pulse are diagnosed as 'cooling'[6]. Weakness, tiredness, cold body temperature and shivering are also reported to be 'cooling' conditions [7].

In agreement with the Chinese belief of yin and yang, health is thought to result from a proper balance of 'heating' and 'cooling' foods and activities, and illness is treated with foods or medicines with properties opposite to those of the disease [8].

Most of foods are neutral. Moreover, foods can change categories as a result of different cooking methods [9]. For example, fried peanuts are typical 'heating' foods, but boiled and steamed peanuts are not. Fried foods and meat cooked in black sesame oil with ginger are also good examples of 'heating' foods produced by a cooking method.

Most 'cooling' foods are of plant origin, especially fruits and vegetables. Many, but not all, of the 'cooling' fruits and vegetables have a high water content, e.g. watermelon and radish, and excretion of a large volume of diluted urine is considered one of the 'cooling' conditions. However, not all vegetables are 'cooling', many are neutral, and a few fruits, such as litchi, longan, and durian, are considered to be 'heating'.

Although there has been speculation on the significance and rationale of the 'hot/cold' food belief from a social science aspect [10], an empirical rational basis has not been reported. A chemical feature common to 'hot' or 'cold' foods cannot be ruled out simply based on the nutrient composition, implying that some components with special functions may be involved. On the other hand, the class of body regulator that is targeted by 'hot' or 'cold' foods and mediates the diverse 'hot/cold' syndrome must be one that has a wide range of functions or can trigger diverse physiological changes in the body.

Since the extreme case of a 'heating' condition resembles inflammation in certain ways, PGE_2 _and NO could be used as the research target for 'heating' foods, feeding mice a diet containing frying oil tended to increase PGE_2 _production by peritoneal macrophages and fried foods are very common 'heating' foods [8,11], the effect of some typical 'heating' and 'cooling' foods on PGE_2 _production was tested using a murine macrophage cell line, J774, as an in vitro model [12]. For example, Lii CK found the ethanol extract of bitter gourd showed the greatest reduction of PGE_2 _production in LPS-stimulated RAW 264.7 macrophage cells [13] and Wu WH et al. identified short and medium chain fatty acids are the major activity ingredient in bitter gourd, in which capric acid exhibited the highest effect [14].

*Litchi chinensis *Sonn (Sapindaceae) is an evergreen shrub or tree, as an improtant economic fruit, is widespread in the south of China (Gontier et al., 2000). As describe above, Litchi is typical a 'heating' fruits which causes inflammatory conditions [15] and there are no reports of specific ingredients found in litchi fruit which take the effects on PGE_2 _or NO production. Therefore, the objective of this study was carried out for the first time to isolate the possible compounds in litchi fruit and to determine their inflammatory effects on PGE_2 _and NO production in J774 murine macrophage cells as well as the related gene expression.

## Materials and methods

### Plant materials

'Huaizhi', the major plant variety of *Litchi chinensis *in Guangdong province were purchased from Guangzhou city Guangdong Province, China, in July 2010. A voucher specimen (NO 20100709) has been deposited at the refrigeration house (-20°C), Faculty of Horticulture, South China Agricultural University.

### Cell culture

J774 murine macrophage cells (originally from the American Type Culture Collection, designation, TIB-67) was obtained from the cell bank of the College of Guangzhou traditional medicine, were cultured in DMEM medium containing 10% heat-inactivated FBS. Cells were incubated at 37°, 5% CO_2 _in a humidified atmosphere.

### PGE_2 _assay

To evaluate the effect of extracts and isolated compounds on PGE_2 _production. Cells seeded (0.5 ml) in 24-well plates at a concentration of 1 × 10^6 ^cell/ml with extracts or the isolated compounds in an incremental concentrations for diluted in the medium, and the maximal concentration that did not significantly affect cell viability were determined by the MTT assay [16]. After 24 h of incubation, the medium were collected and analyzed by HPLC method. HPLC. Waters 2515 system, equipped with a UV detector (waters 2414) at 210 nm, and an Ultimate^® ^XB - 18 column (5 μm, 250 mm × 4.6 mm) obtained from welch materials, Inc. A flow rate 1.0 ml/min was used with two mobile phases acetonitrile and potassium dihydrogen phosphate solution (0.02 mol/L), isocratic elution as a 60 : 40 proportion (v : v).

### Nitrite assay

To evaluate the effects of isolated compounds on NO production, J774 cells seed in 24-well plates were treated with the isolated compounds diluted in the medium in the presence of the same concentration in the PGE_2 _assay. After 24 h of incubation, the medium were collected and analyzed by the Griess reaction [17].

### Extraction

The Litchi fruits (6.6 kg) were dried in shade and the husks were removed from pulp and finely crushed (2.741 kg). The powdered material was extracted with EtOH three times (3 × 25 L) at room temperature, for 5 days each time. The combined EtOH extracts were filtered and evaporated under reduced pressure (room temperature) to give a crude extract (150 g). The extract was suspend in H_2_O (1.5 L), fractionated using a series of organic solvents of increasing polarities, hexane (3 × 4.5 L), AcOEt (3 × 4.5 L) and BuOH (3 × 4.5 L) and then evaporated to dryness using a rotary evaporator (RE-52A, Woshi Co., Shanghai, China) and lyophilized using a freeze-dryer (Freezone plus 12, Labconco, USA) to obtain four different freeze-dried extracts, namely hexane extract (HE), AcOEt extract (EAE), BuOH extract (BE) and water extract (WE).

### Isolation and identification

Column chromatography (CC). Silica gel H (200-300 meshes, Qingdao Marine Chemical Group Co., Ltd. China), visualization under UV light (254 and 365 nm). HPLC separations were performed on a Waters PrepLC system, equipped with a UV detector (waters 2414) at 210 nm, and a X bridge^™ ^Prep C_18 _(5 μm, 150 mm × 10 mm) column, MeOH/H_2_O 80 : 20, 1 ml/min). NMR analysis. The structures of isolated compounds were elucidated by spectroscopic methods including 1D NMR (The NMR spectra were recorded on Bruker-ARX-400 spectrometer (400 MHz for ^1^H and 100 MHz for ^13 ^C), residual solvent peaks as internal standard. Optical rotations: WZZ-2B polarimeter (cell length 1.0 dm, Shanghai Precision Instruments Co., Ltd.). The EAE was subjected to silica gel column chromatography eluting sequentially with petroleum/AcOEt (1 : 20 and 1 : 10) to yield frs. I (10 μl), II (20 μl), and III (15 μl). MeOH/CHCl_3 _(1 : 10, 1 : 9 and 2 : 8) to yield frs. IV (113 mg), V (1.567 g), VI (0.4 g). and frs. VII. Frs. VII was further purified by preparative HPLC (MeOH/H_2_O 80 : 20, 1 ml/min) to give frs. VII-1 (122 mg) and VII-2 (10 μl). frs. IV, V and VII-1 were repeatedly recrystallisation from AcOEt/MeOH to gave crystal (14 mg, 4 mg and 27 mg, resp) which were removed by filtration.

### RT-PCR

Total RNA was extracted from cells in 100-mm dishes using isogen reagents (Nippon Gene Co.). The RNA (3 mg) was subjected to RT-PCR for the measurement of COX-2 and iNOS, and murine GAPDH mRNAs in J774 cells. Total RNA (3 μg) was reverse-transcribed using oligo-(dT)15 primers and avian myeloblastosis virus reverse transcriptase (Promega, Madison, WI, USA). PCR was performed in a reaction mixture containing the obtained cDNA, 0.2 mm dNTP mixture (Promega), 10 pmol of target gene-specific primers, and 0.25 units of Taq DNA polymerase (Promega) using a GeneAmp PCR system 2400 (Applied Biosystems, Foster, CA, USA). Forward and reverse primers for COX-2 were 5'-CCATGTCAAAACCGTGGTGAATG-3' and 5'-ATGGGAGTTGGGCAGTCATCAG-3', resp, 374 bp. Forward and reverse primers for iNOS were 5'-ATGCCCGATGGCACCATCAGA-3' and 5'-CACTTCCTCCAGGATGTTGTA-3', resp, 372 bp. Forward and reverse primers for GAPDH were 5'-CGGAGTCAACGGCTTTGGTCGTAT-3' and 5'-AGCCTTCTCCATGGTGGTGAAGAC-3', resp, 306 bp. All PCR primers used for cDNA were from Clontech Laboratories Inc. Amplifications were performed at 94° for 35 s, at 60° for 2 min and 72° for 2 min with 30 cycles. The amplified PCR products were analyzed on ethidium bromide substituent-stained 1% agarose gels. We confirmed that amplification of COX-2, iNOS, and GAPDH mRNA was linear for 30 cycles. PCR Products were separated by 2% agarose-gel electrophoresis, stained with SYBR-Gold (Molecular Probes, Eugene, Oregon, USA), and visualized by UV transillumination Bio-RAD (Japan). Gene expression was quantified by densitometric scanning using an Image Analyzer Quantity One Installer (Fuji Film Japan). The signal intensities of the specific mRNAs were normalized by comparison with that of GAPDH and were calculated as relative amounts.

### Western blot analysis

Cells were washed twice with cold PBS and were harvested in 150 μL lysis buffer containing 10 mM Tris-HCl, 5 mM EDTA, 0.2 mM phenylmethylsulfonyl fluoride (PMSF), and 20 μg/mL aprotinin, pH 7.4. The protein content in each sample was quantified by use of the Coomassie Plus Protein Assay Reagent Kit (Pierce Chemical Co., Rockford, IL). Equal amounts of proteins were denatured and separated on SDS-polyacrylamide gels and were then transferred to polyvinylidene difluoride membranes (New^™ ^Life Science Product, Inc., Boston, MA). Nonspecific binding sites on the membranes were blocked with 5% nonfat dry milk in a buffer containing 10 mM Tris-HCl and 100 mM NaCl, pH 7.5, at 4°C overnight. The blots were then incubated sequentially with primary antibody (anti- IκBβ and anti-p50 antibodies were purchased from Santa Cruz Biotechnology) and horseradish peroxidase-conjugated anti-mouse IgG (Bio-Rad, Hercules, CA). Immunoreactive protein bands were developed by using 3-3'-diaminobenzidine color developing solution or enhanced chemiluminescence kits (Amersham Life Sciences, Arlington Heights, IL) and then were quantified through densitometric analysis by Zero-Dscan (Scanalytics Inc., Fairfax, VA).

### Statistical analysis

All results were expressed as the mean ± SD for triplicate wells in repeated experiments. The significance of differences at each sample concentration was analyzed by ANOVA and Duncan's multiple range test using PASW 18 software (PASW, Inc., Chicago, IL, USA).

## Results

### Extraction yield and activity evaluation

**Figure 1:**
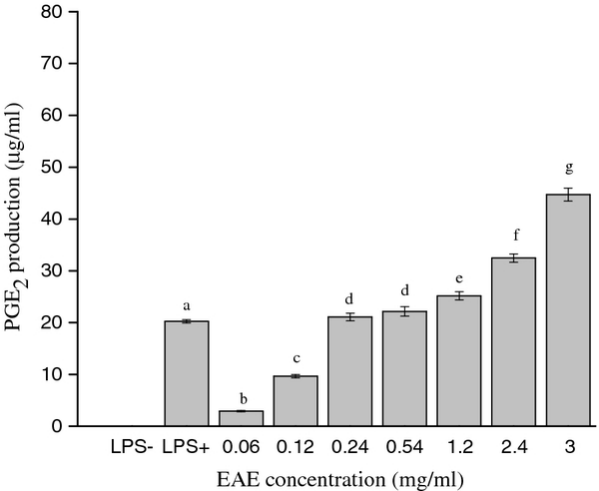
**Concentration-dependent inducing PGE_2 _release in the J774 cells**. Cells were treated with incremental concentrations of the EAE for 24 h, in the presence of 10 μg/ml LPS as the control group. After incubation, the PGE_2 _accumulation in the medium was determined by the HPLC method. Means not sharing a common letter (**a**-**g**) were significantly different (*p *< 0.05) when analyzed by ANOVA and Duncan's multiple range test.

The powdered material of dried litchi was extracted with EtOH, combined EtOH extracts were filtered and evaporated under reduced pressure and the extract was suspend in H_2_O, then fractionated using a series of organic solvents of increasing polarities, hexane, AcOEt and BuOH, evaporated and lyophilized to obtain four different freeze-dried extracts. The BuOH extract (BE) gave the highest extraction yield 98 g, secondly water extract (WE) 43 g, hexane extract (HE) 3.849 g and AcOEt extract (EAE) 3.484 g. J774 cells were incubated with incremental concentrations of each extract for 24 h, and the PGE_2 _accumulation in medium was measured by HPLC method. Results indicated that the EAE exhibited good induce ability in PGE_2 _production, and BE, WE and HE did not (the data were not shown). As shown in Figure 1. PGE_2 _production of the control group by nonactivated cells (without LPS or any other stimulation) was undetected. PGE_2 _production of the LPS group was 20.230 ± 0.320 μg/ml. The EAE caused PGE_2 _release in J774 cells in a dose-dependent manner (PGE_2 _production was at the range of 2.902 ± 0.120-44.711 ± 1.240 μg/ml). At a concentration of 3 mg/ml, the EAE resulted more than 2-fold increase compared with the LPS group.

### Isolation and identification

**Figure 2:**
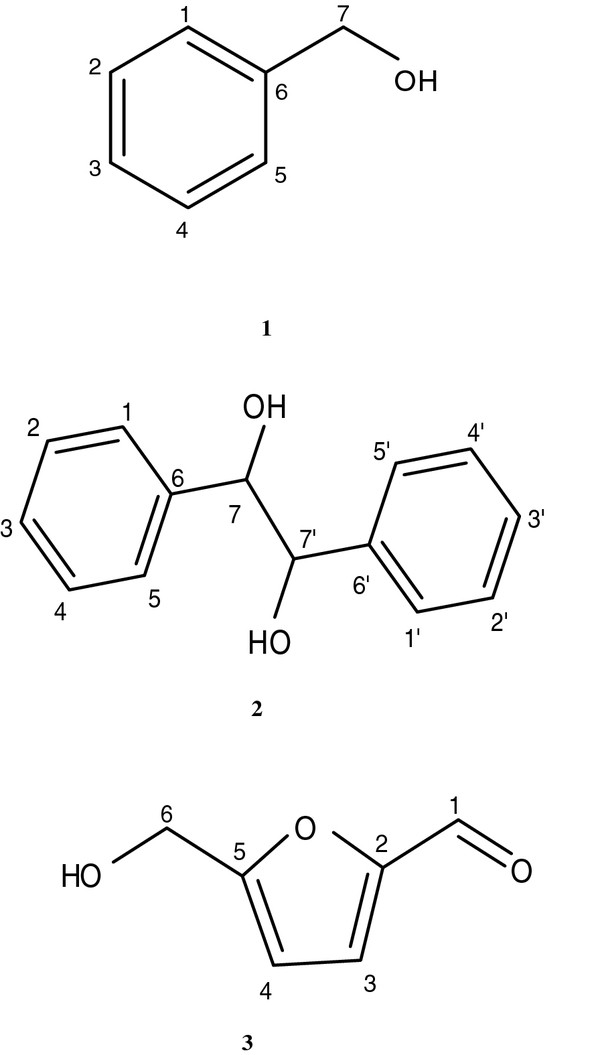
**Structures of Compounds 1-3**.

Seven fractions isolated from the EAE, only frs. III (15 μl), VII-1 (122 mg) and VII-2 (10 μl) were found effective through the activity evaluation experiments on PGE_2 _production in J774 cells, and others did not (the data were not shown). Compound 1 from frs. III was obtained as colorless oil and identified as benzyl alcohol (Figure 2 and Table 1) by comparison of its ^1^H- and ^13 ^C-NMR data with literature values [18]. Compound 2 from frs. VII-1 was obtained as colorless crystal and identified as hydrobenzoin (Figure 2 and Table 1) by comparison of its ^1^H- and ^13 ^C -NMR data with literature values [19]. and the spectra of similar compounds [20]. ${\left[\text{a}\right]}_{\text{D}}^{\text{27}}$= 0 (c = 0.10 MeOH). Compound 3 from frs. VII-2 was obtained as dark color liquid and identified as 5-HMF (Figure 2 and Table 1) by comparison of its ^1^H- and ^13 ^C-NMR data with literature values [21]. Compounds 2-3 were isolated for the first time from the pulp of *Litchi chinensis*.

**Table 1:** NMR Spectroscopic Data for Compounds 1-3.

position	Compound 1		Compound 2		Compound 3	
	
	δ(H)	δ(C)	δ(H)	δ(C)	δ(H)	δ(C)
CH(1,1') or CHO	7.34 (m, 1H)	126.9(s)	7.12 (q, *J = *7.4, 2H)	126.9(s)	9.52 (s, 1H)	179.4(s)

CH(2,2') or C(2)	7.33 (m, 1H)	128.4(s)	7.22 (m, 2H)	128.1(s)		153.9(s)

CH(3, 3')	7.28 (m, 1H)	127.5(s)	7.23 (m, 2H)	127.9(s)	7.37 (d, *J = *3.545, 1H)	124.8(d)

CH(4, 4')	7.33 (m, 1H)	128.4(s)	7.22 (m, 2H)	128.1(s)	6.57 (d, *J = *3.560, 1H)	110.8(d)

CH(5, 5') or C(5)	7.34 (m, 1H)	126.9(s)	7.12 (q, *J = *7.4, 2H)	126.9(s)		163.2(s)

C(6,6') or CH_2_(6)		140.8(s)		139.8(s)	4.60 (s, 2H)	57.6(t)

CH_2_(7) or CH(7)	4.65 (s, 2H)	65.2(s)	4.71 (s, 2H meso)	79.1(d)		

OH	1.98 (br s, 1H)		2.85 (br s, 2H)			

δ in ppm, J in Hz

Compound 1 and Compound 2 Measured in CDCl_3_, Chemical shifts referenced to residual CDCl_3 _(7.256 ppm). Compound 3 Measured in CD_3_OD, Chemical shifts referenced to residual CD_3_OD (49.00 ppm)

### Bioactivity evaluation of the isolated compounds 1-3

**Figure 3:**
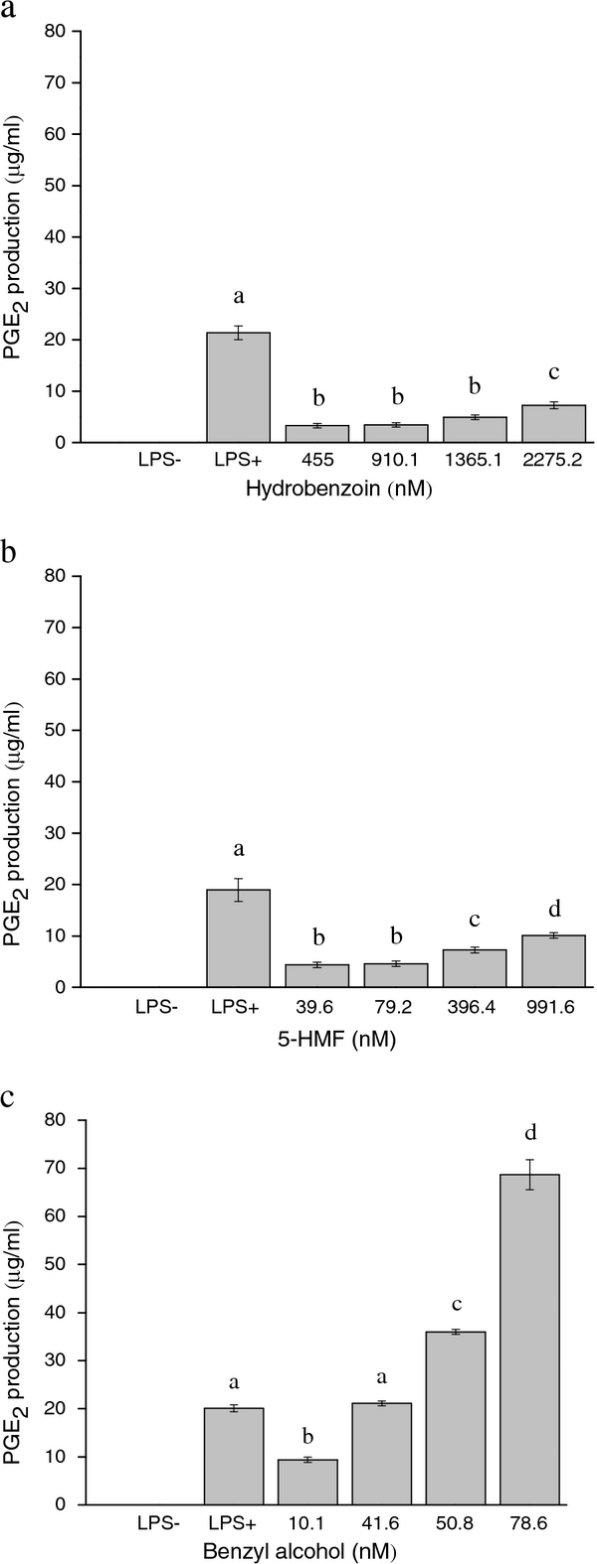
**Concentration-dependent inducing PGE_2 _release in the J774 cells**. Cells were treated with various concentrations of hydrobenzoin, 5-HMF and benzyl alcohol for 24 h, in the presence of 10 μg/ml LPS. After incubation, the PGE_2 _accumulation in the medium was determined by the HPLC system. All the PGE_2 _production data of samples above were within the detect range of HPLC method (0.1448-90 μg/ml). The samples over the range were diluted until appropriate to injecting into HPLC system.

**Figure 4:**
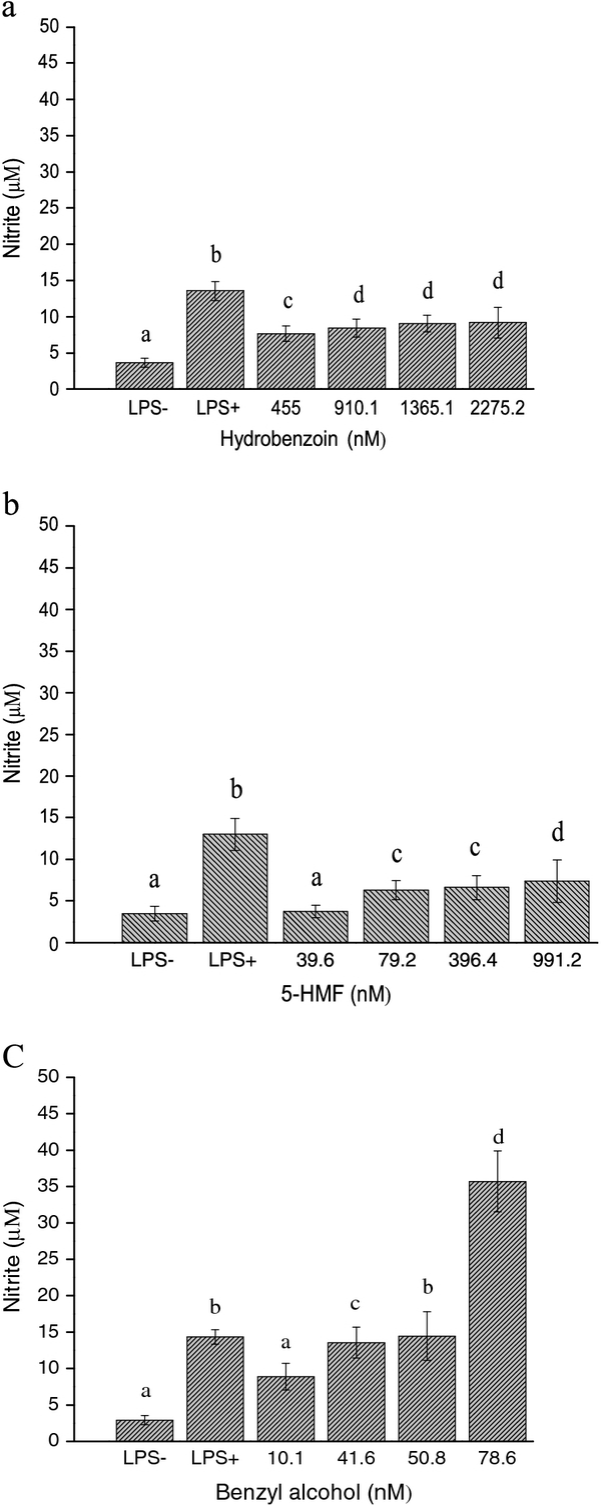
**Concentration-dependent inducing NO release in the J774 cells**. Cells were treated with various concentrations of hydrobenzoin, 5-HMF and benzyl alcohol for 24 h, in the presence of 100 ng/ml LPS. After incubation, the NO accumulation in the medium was determined by the Griess reaction. Means not sharing a common letter (**a**-**d**) were significantly different (*p *< 0.05) when analyzed by ANOVA and Duncan's multiple range test.

As shown in Figure 3, the LPS group caused PGE_2 _release from J774 cells as expected, PGE_2 _production was at the range of 18.875 ± 0.136-23.123 ± 0.123 μg/ml. Hydrobenzoin and 5-HMF moderately stimulated the PGE_2 _production in a dose-dependent manner (at the range of 3.324 ± 0.235-7.230 ± 0.456 and 4.345 ± 0.236-10.098 ± 0.442 μg/ml, resp, Figure 3a-b). Benzyl alcohol caused the greatest PGE_2 _release in a dose-dependent manner (at the range of 9.374 ± 0.234-68.670 ± 3.121 μg/ml, Figure 3c). At a concentration of 78.6 nM, benzyl alcohol resulted in 3.4-fold increase compared with the LPS group. As shown in Figure 4. The three isolated compounds enhanced NO production in a dose-dependent manner without affecting cell viability. Among the three isolated compounds, Benzyl alcohol showed the most potent induce inflammatory activity, the NO production was at the range of 8.867 ± 1.823-35.715 ± 4.191 μM (Figure 4c). The highest concentration of 78.6 nM, resulted in a 2.5-fold increase compared with the LPS group. Hydrobenzoin and 5-HMF also exhibited gentle effects of inducing NO increase, the NO production were at the range of 7.660 ± 1.323-9.168 ± 2.154 and 3.739 ± 0.745-7.359 ± 2.525, resp, μM, (Figure 4a-b).

### COX-2 and iNOS mRNA expression

**Figure 5:**
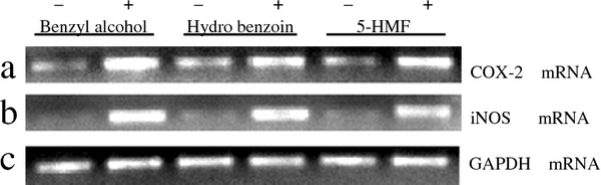
**Effects of the isolated compounds on the expression of COX-2 and iNOS mRNA in J774 cells**. Cells seed in 100-mm dishes were treated with benzyl alcohol (78.6 nM), hydrobenzoin (2.27 μM), and 5-HMF (0.99 μM) respectively for 24 h, (-) means control group and (+) means add sample. Total RNA (3 μg) extracted with isogen reagents was subjected to RT-PCR for the amplification of COX-2 (**a**), iNOS (**b**), GAPDH (**c**) mRNA as described in the text. COX-2 and iNOS mRNA signal intensity was normalized to GAPDH mRNA expression in same sample.

Further to investigate the underlying mechanism of action of cause PGE_2 _and NO release, J774 cells were treated with the maximum effective concentration of benzyl alcohol, hydrobenzoin and 5-HMF respectively for 24 h. RNA were obtained and further analyzed by RT-PCR. As shown in Figure 5, we found very moderate expression of COX-2 and no iNOS mRNA in non-activated cells. Benzyl alcohol selectively upregulate expression of the COX-2 and iNOS transcript markedly, and the COX-2 and iNOS mRNA expression of the J774 cells activated by hydrobenzoin and 5-HMF were also increased. The results indicated the regulation of COX-2 and iNOS mRNA expression might be associated with the stimulative effect of the three isolated compounds on PGE_2 _and NO production.

### NF-κB activation

**Figure 6:**
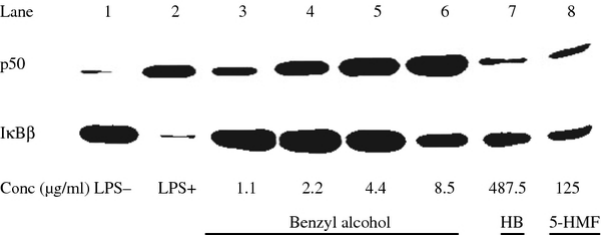
**Effects of the iaolated compounds on NF-κB activation**. Cells were treated with various concentrations of benzyl alcohol, the highest effective concentration of hydrobenzoin and 5-HMF for 12 h harvested for western bolt analysis of p50 and IκBβ protein expression. in the presence of 100 ng/ml LPS, six replicates of the same experiment is shown.

We performed a western bolt assay to examine whether the isolated compounds could modulate the inflammatory response by NF-κB activation. As shown in Figure 6, benzyl alcohol-stimulated macrophages showed a marked increase in p50 and decrease in IκBβ in a dose-manner with the concentration (lane 3-6), compared with the unstimulated macrophages and LPS-stimulated macrophages (lane 1 and 2). At the highest effective concentration, hydrobenzoin and 5-HMF also stimulated macrophages a increase in p50 and decrease in IκBβ in varying degrees (lane 7 and 8).

## Discussion

COX-2, iNOS expression is induced in macrophages in response to intrinsic factors such as cytokines, or extrinsic factors such as LPS, leading to the production of PGE_2_, NO [22,23]. Most of these factors are molecules that can trigger the signaling pathway by binding to receptor molecules in the cell membrane. This activates the signal transduction pathway, which includes protein kinase C and MAPK [24,25]. Expression of the COX-2 or iNOS gene is induced when these signals activate transcriptional factors, such as NF-κB [25,26]. Theoretically, molecules able to bind to cell surface receptors, trigger the signaling pathway, or activate transcription factors could directly or indirectly induce COX-2 or iNOS protein expression and result in PGE_2 _and NO production. There should be a further experiment such as western blot to identify the signal transduction pathway and make sure the detailed mechanism of effects of Litchi Fruit Isolates in J774 murine macrophage Cells

In conclusion, our results demonstrated that *Litchi chinensis *fruit bear potent activities of induce PGE_2 _and NO increase. The data of effects of hydrobenzoin, 5-HMF and benzyl alcohol on PGE_2 _and NO production was coincident with COX-2 and iNOS mRNA expression, and the NF-κB is the possible molecular machanism. This study will provide the basic components information supporting further well-controlled in vivo experiments and mechanism of action, and hope to partially explain the 'heating' in traditional Chinese medicine theory.

## Abbreviations

DMEM: Dulbecco's modified eagle's medium; EAE: Ethyl acetate-soluble part of an ethanol extract; PGE_2_: Prostaglandin E_2; _NO: Nitric oxide: LPS; Lipopolysaccharide; 5-HMF: 5-hydroxymethyl-2-furfurolaldehyde; COX-2: Cyclooxygenase 2; HB: Hydrobenzoin.

## Competing interests

The authors declare that they have no competing interests.

## Authors' contributions

Yang Zhou carried out the most of the studies, specially participated in Extraction, Isolation and identification. Hong Wang participated in Nitrite assay. Ruili Yang participated in Western blot analysis. Hui Huang, Yudong Shen participated in PGE_2 _assay. Hongtao Lei participated in RT-PCR. Hong Gao participated in Cell culture. Yuanming Sun and Yang Zhou conceived of the study, and participated in its design and coordination. All authors read and approved the final manuscript.

## Pre-publication history

The pre-publication history for this paper can be accessed here:

http://www.biomedcentral.com/1472-6882/12/12/prepub
